# Comparison of clinical efficacy between proximal femoral locking plate and cannulated compression screws for femoral neck fracture

**DOI:** 10.3389/fsurg.2025.1546873

**Published:** 2025-04-10

**Authors:** Xianglong Zhou, Dongxuan Wei, Jiheng Xiao, Tianyi Xia, Haoran Zhou, Jianhui Xiang, Hanhong Fang, Hui Song, Liming Xiong

**Affiliations:** Department of Orthopaedics, Union Hospital, Tongji Medical College, Huazhong University of Science and Technology, Wuhan, China

**Keywords:** hip fracture, femoral neck fracture, internal fixation, proximal femoral locking plate, cannulated compression screws

## Abstract

**Objective:**

The objective of this study is to investigate the clinical efficacy of proximal femoral locking plates in comparison to cannulated compression screws for the treatment of femoral neck fractures.

**Methods:**

A retrospective analysis of clinical data from 50 patients with femoral neck fractures treated at the Department of Orthopaedics, Union Hospital, Tongji Medical College, Huazhong University of Science and Technology from September 2018 to June 2023. Patients were divided into 25 in the PFLP group and 25 in the CCS group. A comparison was made between the two groups in terms of the following variables: basic characteristics, perioperative information, and information during follow-up visits.

**Results:**

The basic characteristics of the two groups were found to exhibit no statistically significant differences (*P* > 0.05). The PFLP group had significantly longer surgical times and greater Intraoperative hemorrhage loss compared to the CCS group (*P* < 0.05). The statistical analysis revealed that there were no significant differences in intraoperative fluoroscopy times and Garden index between the two groups (*P* > 0.05). The PFLP group exhibited a markedly superior fracture healing time, femoral neck shortening, and Harris hip scores in comparison to the CCS group (*P* < 0.05). The postoperative complication rates were 12% in the PFLP group and 20% in the CCS group, with no statistically significant difference (*P* > 0.05).

**Conclusion:**

The results of this retrospective study suggest that the PFLP group demonstrated superior outcomes compared to the CCS group in several key areas, including fracture healing time, preservation of femoral neck length, recovery of hip function, and incidence of postoperative complications.

## 1 Introduction

In recent years, the acceleration of global population aging and the frequent occurrence of traffic accidents have led to a significant increase in the incidence of hip fractures, placing a substantial burden on society and the healthcare system (1, 2). It is predicted that there will be 4.5 million hip fractures worldwide by 2050 (3). Among all types of hip fractures, those affecting the femoral neck are the most prevalent, accounting for 3.6% of all fractures and 50% of hip fractures (4). Femoral neck fractures present a substantial challenge in the field of Orthopaedics, exhibiting a high degree of severity and a multitude of complications. The implementation of conservative therapeutic modalities has been observed to frequently result in elevated rates of disability and mortality (5, 6). According to established principles of treatment for femoral neck fractures, early surgical intervention is recommended for the majority of patients who do not have contraindications to surgery (7–9).

The objective of surgical treatment is to restore stability and functionality to the hip joint, thereby reducing the incidence of complications associated with prolonged bed rest, including deep vein thrombosis, pneumonia, and pressure ulcers (10). Currently, the mainstay of surgical treatment for femoral neck fractures is internal fixation or hip arthroplasty. The specific approach is determined by the patient's age, physical condition and the anticipated functional requirements following the procedure (11). For middle-aged and young patients, as well as some elderly patients who are unable to tolerate arthroplasty and those with minor fracture displacement, internal fixation remains the primary choice and is a safe and effective treatment option (10, 12, 13). The primary advantages of internal fixation of femoral neck fractures include preservation of the femoral head, minimal trauma, and a simple surgical technique. The principal determinant of surgical success is the achievement of good anatomical reduction and stable internal fixation (14, 15). The use of stable internal fixation has been shown to facilitate postoperative recovery and reduce the prevalence of complications (16, 17). The most commonly employed internal fixation methods include the use of cannulated compression screws (CCS), femoral neck systems (FNS), proximal femoral locking plates (PFLP) and the combination of cannulated screws with medial support plates. The various methods have distinct advantages and limitations, and there is currently a debate among experts regarding the optimal internal fixation device for treatment (17, 18). The objective of this study is to investigate the clinical efficacy of internal fixation as a treatment for femoral neck fractures. To this end, a retrospective analysis has been conducted to compare the outcomes of proximal femoral locking plates and cannulated screws, with the objective of providing a reference for the treatment of these fractures.

## 2 Materials and methods

### 2.1 Patients

The clinical data of 50 patients with femoral neck fractures admitted to the Department of Orthopaedics of Union Hospital, Tongji Medical College, Huazhong University of Science and Technology, China, from September 2018 to June 2023 were retrospectively analyzed. Informed consent was obtained from all participants, and ethical approval was granted by the Medical Ethics Committee of Union Hospital, Tongji Medical College, Huazhong University of Science and Technology (Approval No. 20240652).

### 2.2 Inclusion criteria and exclusion criteria

Inclusion criteria: (1) Age 18–65 years (2) Unilateral, closed, fresh femoral neck fracture (less than 3 weeks) (3) Diagnosis of femoral neck fracture based on history, physical examination, and imaging data (4) Normal function of the affected hip joint prior to injury (5) Absence of contraindications to surgery (6) Follow-up of at least 1 year with complete clinical data Exclusion criteria: (1) old, pathological or open femoral neck fractures (2) Serious dysfunction of vital organs such as the heart, lungs, brain and other vital organs, unable to tolerate surgery and anesthesia (3) combined with fracture and dysfunction of other parts of the lower limb (4) poor patient compliance and incomplete clinical data.

### 2.3 Preoperative treatment

All patients were provided with pre-operative symptomatic treatment, including pain relief, edema reduction, and thrombosis prophylaxis. Patients underwent a series of diagnostic imaging procedures, including positive and lateral x-rays, CT scans with three-dimensional reconstruction of the affected hip joint, and ultrasound examinations of the deep and superficial veins of both lower limbs. Pre-operative examinations were performed to exclude contraindications to anesthesia and surgery, and if necessary, cardiovascular medicine, respiratory and critical care medicine, anesthesiology and other related departments were invited to consult with patients to assess their tolerance to surgery.

### 2.4 Surgical method

**Figure 1:**
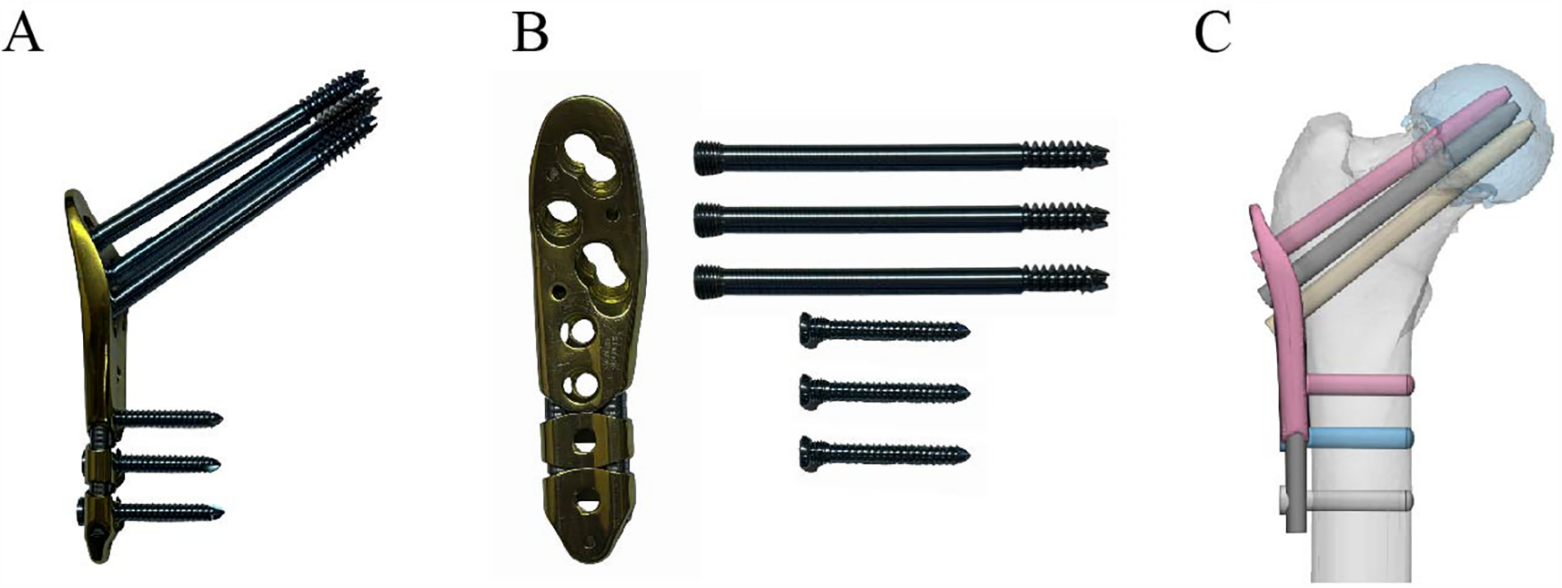
A schematic diagram of a proximal femoral locking plate **(A**,**B)** and the treatment of a femoral neck fracture **(C)**.

**Figure 2:**
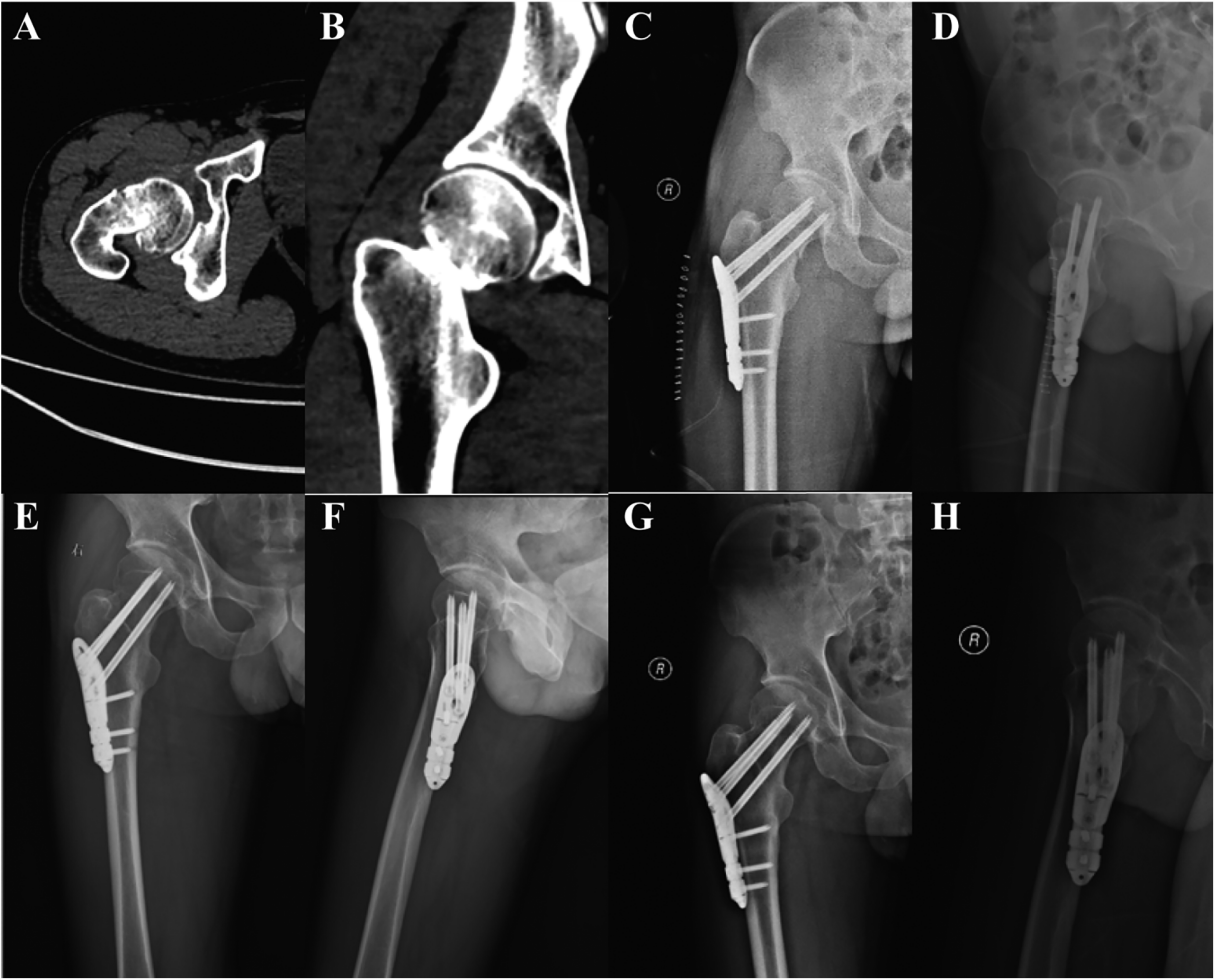
A 40-year-old male patient was admitted to the hospital due to pain and restricted range of motion in the right hip following a traffic injury, and he underwent PFLP treatment. **(A,B)** Pre-operative CT images of the patient showed a right femoral neck fracture, Garden type IV. **(C,D)** Review radiographs 1 day post-operatively in the anteroposterior and lateral positions showed good reduction. **(E,F)** Review radiographs 4 months post-operatively. **(G,H)** Review radiographs 12 months post-operatively.

**Figure 3:**
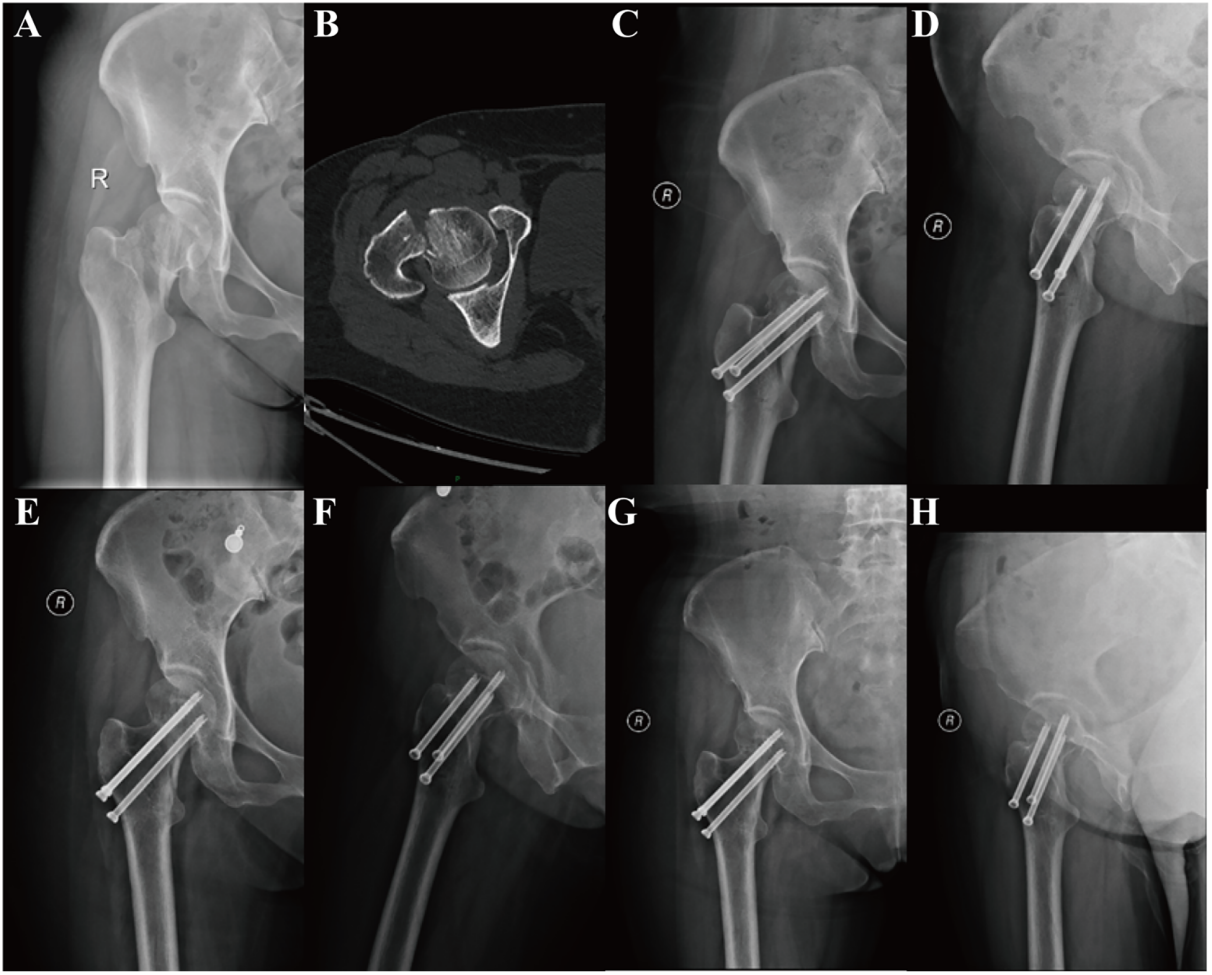
A 50-year-old female patient was admitted to the hospital for treatment of right hip pain and limited range of motion following a fall and underwent CCS treatment. **(A,B)** Pre-operative CT images of the patient showed a right femoral neck fracture, Garden type IV. **(C,D)** Review radiographs 1 day post-operatively in the anteroposterior and lateral positions showed good reduction. **(E,F)** Review radiographs 4 months post-operatively. **(G,H)** Review radiographs 13 months post-operatively.

All patients were administered general anesthesia with tracheal intubation. The patients were positioned in the supine position on an orthopedic lower extremity traction bed. Following the satisfactory completion of closed reduction, the traction bed was secured, and the standard disinfection and draping procedures were conducted. PFLP group (Figure 1): A straight lateral incision of approximately 5 cm was made distally, starting from the highest point of the greater trochanter of the femur. The tissue was then dissected layer by layer until the lateral cortex of the femur was exposed. A proximal femoral locking plate (PFLP) of appropriate length (Tianjin Weiman Biomaterials Company) was placed on the proximal femur. A guide pin was inserted, the depth of the guide pin was measured with a reamer, cannulated locking screws were sequentially inserted, and the position and depth of the cannulated screws were verified with C-arm fluoroscopy. Subsequently, the guide pin was extracted. The screws were positioned within the distal femoral shaft region of the proximal femoral locking plate for fixation. A typical case is shown in Figure 2. CCS group: An inverted triangular configuration was utilized to insert guide pins percutaneously. Subsequent adjustments were made to the position and depth of the guide pins under C-arm fluoroscopy in both anteroposterior and lateral views. The depth of the guide pins was determined by means of a reamer, and cannulated screws (Wuhan Mindray Company) were then inserted in a sequential manner. In patients with relatively weak lateral femoral cortices, the addition of supplementary washers may be considered. Subsequently, the guide pin was extracted. A typical case is shown in Figure 3. The incision was irrigated with saline solution, then sutured in layers and covered with a sterile dressing.

### 2.5 Postoperative management

In the postoperative period, the patient's vital signs were monitored, and pain relief and prophylactic antibiotic therapy were administered in accordance with standard practice. A postoperative review of the hip positive and lateral radiographs was conducted. Following surgery, patients were instructed to perform passive flexion and extension of the hip and knee joints of the affected limb, as well as muscle function exercises. The sutures were removed 10–12 days following the surgical procedure. Patients were monitored via outpatient visits and telephone calls.

### 2.6 Observation index and evaluation of curative effect

The study included 50 patients with femoral neck fractures, divided into two groups: the PFLP group (*n* = 25) and the CCS group (*n* = 25). The baseline characteristics included patient age, sex, body mass index (BMI), side of fracture, cause of injury, Garden classification, time from injury to surgery, and duration of follow-up. Perioperative information included Surgical time, intraoperative hemorrhage, intraoperative fluoroscopy times. Subsequent data included a postoperative reassessment of reduction quality (Garden index), fracture healing time, femoral neck shortening at 1 year postoperatively or at the final follow-up, and hip Harris (19) score. The postoperative complications observed included osteonecrosis of the femoral head, bone nonunion, screw withdrawal, and screw breakage.

### 2.7 Statistical analysis

All data analyses were conducted using IBM SPSS Statistics 29.0 software, as specified in the study methodology. The normality of the distribution of the quantitative data was assessed using the Shapiro–Wilk test, while the equality of variances was evaluated through Levene's test. The quantitative results were expressed as mean ± standard deviation. The data indicated that the variable in question exhibited a mean value of $\left(\bar{X}\pm S\right)$. The differences between the two groups were then compared using a two-independent-samples *t*-test. Comparisons between groups of qualitative data were analyzed using the chi-squared test or Fisher's exact probability method. All statistical tests were conducted with a significance level of *P* < 0.05 to ascertain the statistical significance of observed differences.

## 3 Results

### 3.1 Basic characteristics

The differences in age, gender, BMI, fracture side, cause of injury, fracture type, time from injury to surgery, and follow-up time between the two groups were not statistically significant (*P* > 0.05, Table 1), and were comparable.

**Table 1:** Basic characteristics of all patients treated.

Basic characteristics	PFLP patients (*n* = 25)	CCS patients (*n* = 25)	*P* value
Age (years)	50.12 ± 10.27	48.48 ± 10.69	0.54
Sex			0.77
Male (*n*,%)	17 (68%)	16 (64%)	
Female (*n*,%)	8 (32%)	9 (36%)	
BMI	22.46 ± 2.72	22.96 ± 2.91	0.53
Side of fracture			0.78
Left (*n*,%)	10 (40%)	9 (36%)	
Right (*n*,%)	15 (60%)	16 (64%)	0.37
Fall-related injury (*n*,%)	18 (72%)	20 (80%)	
Traffic injury (*n*,%)	7 (38%)	5 (20%)	
Garden classification			0.28
I&II (*n*,%)	8 (32%)	5 (20%)	
III&IV (*n*,%)	17 (68%)	20 (80%)	
Time from injury to surgery (days)	2.96 ± 0.94	2.64 ± 1.25	0.31
Follow-up duration (months)	18.4 ± 2.90	17.48 ± 2.5	0.232

### 3.2 Perioperative information

Patients treated with PFLP exhibited longer surgical times (81.01 ± 6.43 min vs. 69.28 ± 15.93 min) and increased intraoperative hemorrhage (85.47 ± 18.85 ml vs. 36.76 ± 6.64 ml), with a statistically significant difference (*P* < 0.05, Table 2). No statistically significant difference was observed in the number of intraoperative fluoroscopies and fracture reduction Garden index between the two groups (*P* > 0.05, Table 2).

**Table 2:** Perioperative information for all patients treated.

Perioperative information	PFLP patients (*n* = 25)	CCS patients (*n* = 25)	*P* value
Surgical time (min)	81.01 ± 6.43	69.28 ± 15.93	0.01
Intraoperative hemorrhage (ml)	85.47 ± 18.85	36.76 ± 6.64	<0.01
Intraoperative fluoroscopy (times)	10.52 ± 3.08	12.64 ± 4.43	0.55
Garden index			0.284
I (*n*,%)	22 (88%)	21 (84%)	
II (*n*,%)	3 (12%)	4 (16%)	

### 3.3 Information during follow-up visits

The mean fracture healing time for patients in the PFLP group was 4.85 ± 1.10 months, which was significantly shorter than that of the CCS group (5.66 ± 0.71 months; *P* < 0.05, Table 3). Both groups exhibited femoral neck shortening following surgery; however, this was significantly less pronounced in the PFLP group compared to the CCS group (1.09 ± 0.99 mm vs. 4.26 ± 4.49 mm, *P* < 0.05, Table 3). The postoperative Haaris scores for the two groups exhibited a notable disparity (*P* < 0.05, Table 3), with a mean of 89.12 ± 2.13 for the PFLP cohort and a mean of 84.52 ± 8.43 for the CCS group. With regard to postoperative complications, the incidence rates in the two groups were 12% in the PFLP group and 20% in the CCS group, with no statistically significant difference (*P* > 0.05, Table 3).

**Table 3:** Information on the follow-up period for all patients treated.

Follow-up duration information	PFLP patients (*n* = 25)	CCS patients (*n* = 25)	*P* value
Fracture healing time (months)	4.85 ± 1.10	5.66 ± 0.71	0.03
Changes in femoral neck shortening (mm)	1.09 ± 0.99	4.26 ± 4.49	0.01
Harris score at final follow up (or 1 year)	89.12 ± 2.13	84.52 ± 8.43	0.01
Complications	3 (12%)	5 (20%)	0.45
Osteonecrosis of the femoral head	2 (8%)	3 (12%)	
Bone nonunion	1 (4%)	1 (8%)	
Screw withdrawal	0	1	
Screw breakage	0	0	

## 4 Discussion

Surgical treatment is the primary intervention for most patients with femoral neck fractures, with the specific surgical approach often contingent on the patient's age. Currently, arthroplasty is the preferred surgical option for elderly patients, particularly those aged 75 and above (20, 21). Although arthroplasty is a more invasive procedure and is associated with complications such as periprosthetic infection and loosening, it has a shorter treatment period and can rapidly reduce pain, restore hip function, and achieve early weight bearing (22). For patients in the younger and middle-aged demographic, internal fixation remains the primary treatment option (23). Nevertheless, a paucity of evidence-based medical literature exists to define the optimal internal fixation treatment. In the present study, we present the clinical results of treating femoral neck fractures with PFLP and CCS. The results showed that the fracture healing time, degree of femoral neck shortening, and Harris score of patients in the PFLP group were significantly superior to those in the CCS group; however, operative time and intraoperative bleeding were longer in the PFLP group compared to the CCS group. These results indicate that PFLP has the potential to accelerate fracture healing, reduce the extent of postoperative femoral neck shortening, and improve hip functionality, but is associated with increased operative time and intraoperative bleeding, and its efficacy in the remaining observed indicators is comparable to that of CCS.

The PFLP treatment of femoral neck fractures necessitates an incisional approach, which entails a larger incision, augmented exposure, and a longer surgical time frame. The advantages of the PFLP include its conformity to the anatomical design of the proximal femur and its preset collo-diaphyseal angle and angle of inclination (24). The design in question has been demonstrated to reduce the damage to the blood supply of the femoral head that is caused by periosteal stripping and repeated adjustments of the guide pins. It is therefore still in the category of minimally invasive surgery. The PFLP demonstrates notable biomechanical advantages, with the threads of its hollow screw tail locking into the plate as a whole. This transfers the bending force borne by the head-neck screws to the femoral diaphysis cortex, providing favorable angular stability and anti-rotation capability. Furthermore, it allows patients to bear weight and undergo rehabilitation training at an early postoperative stage (25–27). This approach better preserves the length of the femoral neck and reduces the incidence of complications such as screw loosening, screw withdrawal, and screw breakage. As a result, the likelihood of requiring a revision procedure is decreased (28). Although the number of studies on PFLP for femoral neck fractures is limited, the results of the available studies corroborate some of the findings of this study (29–31). Although CCS for femoral neck fractures offers several advantages, including a short operative time, minimal surgical trauma, ease of use, and a high healing rate, its three-screw fixation has insufficient resistance to inversion and shear. This can result in fracture end loosening, which may lead to complications such as screw withdrawal, screw breakage, femoral neck shortening, and secondary subrotator fracture (32, 33).

**Figure 4:**
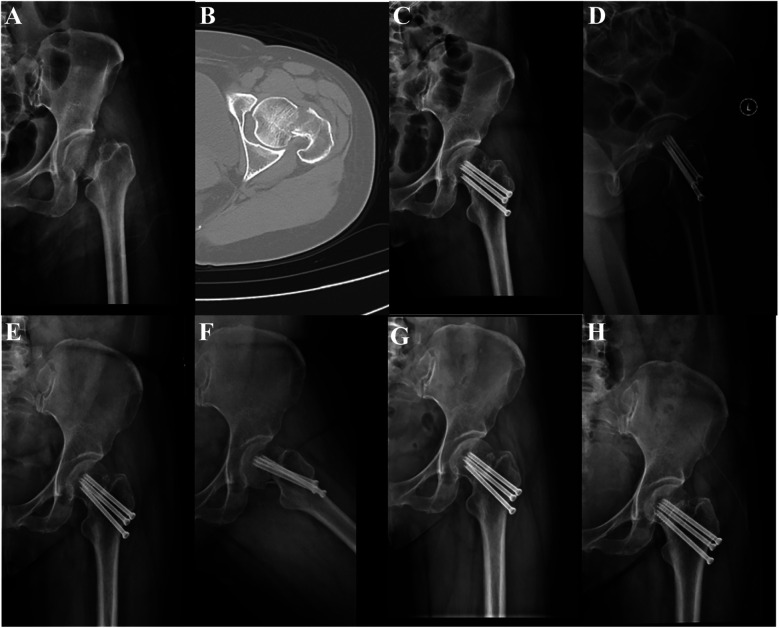
A 50-year-old female patient was admitted to the hospital for treatment of left hip pain and limited range of motion following a fall and underwent CCS treatment. **(A,B)** Pre-operative CT images of the patient showed a left femoral neck fracture, Garden type IV. **(C,D)** Review radiographs 1 day post-operatively in the anteroposterior and lateral positions showed good reduction. **(E–H)** Review radiographs at 12 and 18 months postoperatively showed femoral neck shortening.

One of the keys to the treatment of femoral neck fractures is quality control of the reduction, and poor reduction is an important factor leading to healing complications and reoperation (34). It is also imperative to give due attention to the correct choice of internal fixation and precise screw placement. Adequate reduction facilitates accurate nailing, while stable internal fixation ensures reduction efficiency and motion stability. In order to ensure optimal outcomes, fluoroscopy of the frontal and lateral views is essential during surgery. This allows for a comprehensive assessment of the fracture site, including the line of force, the cervical shaft angle, the anterior tilt angle, the alignment of the cortex, and the position of the screw. The use of hollow screw fixation in the treatment of femoral neck fractures has been shown to result in dynamic compression of the fracture end during weight bearing. However, this approach has also been associated with an increased risk of shortening the femoral neck (Figure 4).

**Figure 5:**
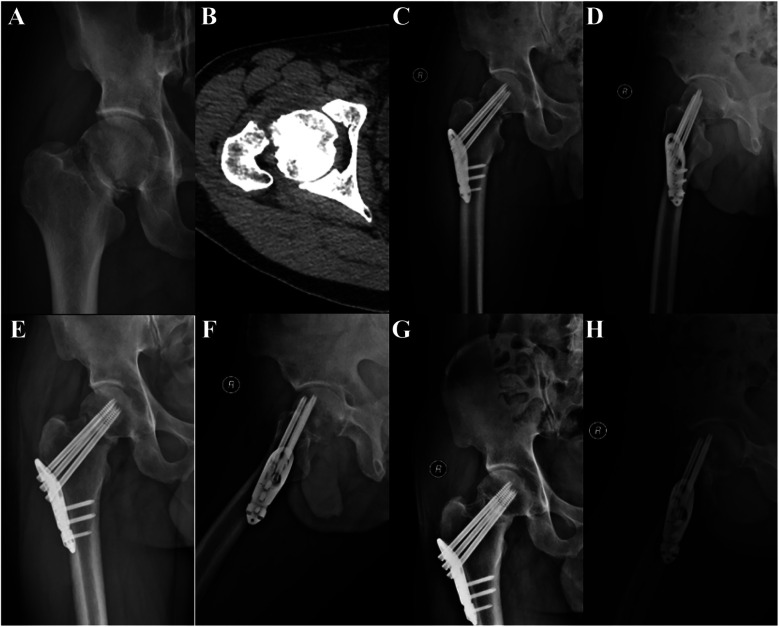
A 52-year-old male patient was admitted to the hospital for treatment of right hip pain and limited range of motion following a fall and underwent PFLP treatment. **(A,B)** Pre-operative CT images of the patient showed a right femoral neck fracture, Garden type IV. **(C,D)** Review radiographs 1 day post-operatively in the anteroposterior and lateral positions showed good reduction, but a gap was present at the fractured end. **(E,F)** Review radiographs 3 months post-operatively showed gradual fracture healing and a reduction in the fracture gap. **(G,H)** Review radiographs 6 months post-operatively showed disappearance of the gap and good fracture healing.

According to the findings of previous studies, femoral neck shortening has been demonstrated to result in diminished gait velocity and to manifest as an impairment in gait symmetry and physical functioning (35). A multicenter cohort study by Zlowodzki et al. (36) further revealed the impact of femoral neck shortening on patients' postoperative quality of life. The study identified significant variations in EuroQol questionnaire scores and SF-36 physical function scores between patients with femoral neck shortening greater than 5 mm and those with shortening less than 5 mm. In a prospective multicenter study of hip fracture prognosis in China, researchers found that femoral neck shortening was significantly associated with poorer hip function in patients under 55 years of age (37). Notably, in that study, 35.7% of patients with femoral neck fractures treated with hollow nails experienced more than 5 mm of femoral neck shortening. A recent study found that the modified Harris Hip Score (mHHS) and Visual Analogue Score (VAS) were significantly lower in the femoral neck shortening group (≥5 mm) than in the no shortening group (38). Finite element analysis revealed that the stress distribution in the hip joint changed after the shortening of the femoral neck, and the stress distribution in the femoral head was not uniform (39, 40). When there is severe shortening of the femoral neck, the unevenness of load distribution is further exacerbated and hip mobility is significantly reduced, which may also lead to an increased probability of femoral head necrosis. The femoral neck is susceptible to postoperative shortening due to its anatomical and biomechanical characteristics. A correlation has been observed between shortening of the femoral neck and the onset of pain and decreased hip function, and this shortening may increase the long-term risk of osteonecrosis of the femoral head. In general, the femoral neck is prone to postoperative neck shortening due to its special anatomical structure and biomechanical properties, and there is a correlation between shortening of the femoral neck and the development of pain and decline in hip function. They may also increase the long-term risk of femoral head necrosis. The findings of our study demonstrated that the PFLP group exhibited a notable advantage over the CCS group with respect to the preservation of femoral neck length and postoperative Harris scores. This may be attributed to the enhanced mechanical stability and shear resistance of the PFLP plate screw locking structure (41). Furthermore, in our case, we found that although PFLP did not have the effect of CCS sliding compression, fracture healing could still be achieved with good repositioning and strong internal fixation, even if there was a gap at the fracture break (Figure 5).

In this study, the PFLP group achieved relatively satisfactory treatment results. For middle-aged patients younger than 60 years and stable fracture patients between 60 and 75 years, PFLP can be an effective treatment for femoral neck fractures if they are in good physical condition, have a high activity level and a strong will to preserve the hip (9). Nevertheless, the practice of internal fixation of femoral neck fractures continues to present a number of significant challenges. First, internal fixation requires a long recovery period, and fracture nonunion and femoral head necrosis are always insurmountable obstacles. In addition, repeated nailing adjustments during surgery may exacerbate bone loss, especially in osteoporotic patients. Therefore, it is imperative that the treatment cycle and risk of secondary revision surgery be adequately considered when performing internal fixation of femoral neck fractures. When formulating an individualized treatment strategy, it is important to consider the patient's age, general condition, current mobility, and anticipated functional needs, as well as the indications for surgery. It is imperative to assess the immediate and long-term consequences of disparate treatment modalities on the patient in order to select the most suitable treatment approach and achieve the optimal outcome.

This study is a single-center retrospective cohort study with a low level of evidence in evidence-based medicine. As a result, there is a possibility of selection bias during the study. Furthermore, the sample size of this study is relatively small, the follow-up period is relatively brief, and the postoperative follow-up period may be insufficient to fully observe the occurrence of femoral head necrosis and other complications. It would be beneficial for future research to focus on conducting large prospective multicenter randomized controlled trials, which would allow for a more comprehensive and detailed evaluation of the efficacy of treatment modalities for femoral neck fractures.

## 5 Conclusion

In conclusion, both the PFLP and CCS groups achieved satisfactory efficacy in the treatment of femoral neck fractures. Compared with the CCS group, the PFLP group showed more significant advantages in fracture healing time, preservation of femoral neck length and restoration of hip function. Therefore, PFLP achieved satisfactory results in the treatment of femoral neck fractures and can be used as an effective internal fixation method for the treatment of femoral neck fractures.

## Data Availability

The original contributions presented in the study are included in the article/Supplementary Material, further inquiries can be directed to the corresponding author.
